# Structural Constraints on the Covariance Matrix Derived from Multiple Aligned Protein Sequences

**DOI:** 10.1371/journal.pone.0028265

**Published:** 2011-12-05

**Authors:** William R. Taylor, Michael I. Sadowski

**Affiliations:** Division of Mathematical Biology, MRC National Institute for Medical Research, London, United Kingdom; Cardiff University, United Kingdom

## Abstract

Residue contact predictions were calculated based on the mutual information observed between pairs of positions in large multiple protein sequence alignments. Where previously only the statistical properties of these data have been considered important, we introduce new measures to impose constraints that make the contact map more consistent with a three dimensional structure. These included global (bulk) properties and local secondary structure properties. The latter allowed the contact constraints to be employed at the level of filtering pairs of secondary structure contacts which led to a more efficient (lower-level) implementation in the PLATO structure prediction server. Where previously the measure of success with this method had been whether the correct fold was predicted in the top 10 ranked models, with the current implementation, our summary statistic is the number of correct folds included in the top 10 models — which is on average over 50 percent.

## Introduction

The compact nature of the folded protein chain imposes constraints on the mutational freedom of packed pairs of residues. However, unlike the more rigorous 1∶1 base-pairing of nucleotide interaction found in RNA, the constraints on amino acid interactions are less specific and the effect on any one position is an average over contributions from all its neighbouring residues. This results in a weak signal that barely exceeds background ‘noise’ effects [1] and over the years many approaches have been used to try and tease-out useful structural constraints from the noise [2], [3], [4].

In recent years, fast sequencing methods have led to an explosion of sequence data from a diverse range of organisms and the analysis and alignment of these sequences have identified many protein families with several thousand members [5]. Using these large sequence families, it now appears that the information in pairwise residue correlations is approaching a threshold at which useful structural constraints can be obtained [6]. In that work, we showed that predicted contacts derived from processing the mutual information (MI) between positions in a multiple sequence alignment could be used to select the correct fold from a large collection of well constructed decoys. This was achieved by re-scoring the models using the direct contact (DC) predictions extracted from the MI values. However, this approach relied on the decoy generation method (PLATO) to create a model of the correct fold and sufficient variations of it to allow the DC values to be matched to the correct residue pairs.

In this work, we follow a similar approach but instead, shift the application of the DC constraints to a deeper level in the PLATO method to filter the generation of the folds — keeping only those folds that are compatible with the predicted contacts for construction at the α-carbon (residue) level. This lower level application at a more symbolic level of secondary structures element (SSE) representation required the development of a new scoring scheme that predicts the polarity (parallel/antiparallel) of the SSE pairs. In addition we also apply some new filters at the residue level to re-balance the predicted contacts towards distributions that are more compatible with typical globular domains.

## Results

### Residue packing analysis

#### Analysis of SCOP40

**Figure 1:**
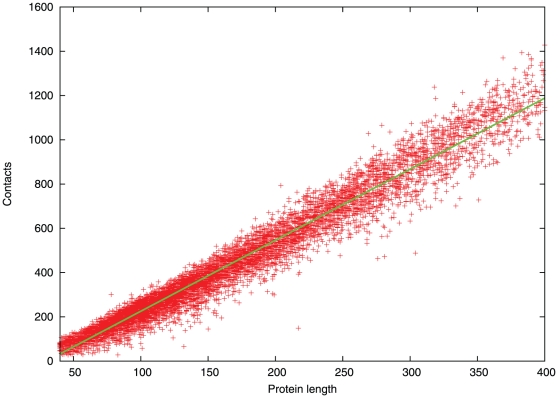
Number of contacts with protein length. The number of contacts between pseudo-centroids for the domains included in the SCOP40 database is plotted against the number of residues in the domain (Protein length). The green line is the best-fit to the data over the plotted range.

The number of contacts under 8 Å expected between pseudo-centroids for proteins of different size was estimated from their distribution over the SCOP40 database. [7] (Figure 1).

It can be seen from Figure 1 that the increase in the number of contact residues is directly proportional to the size of the protein and can be effectively modelled with a linear fit, which over the (domain size) range of the data plotted in Figure 1, was: *P* = 3.21*N*-95.7, where *N* is the number of residues in the protein and *P* the estimated number of packed pairs. It can also be seen from Figure 1 that the spread of values does not increase markedly with length, with a standard deviation of *P*+/−50 being a reasonable approximation over the range of proteins considered here (100–200 residues).

**Figure 2:**
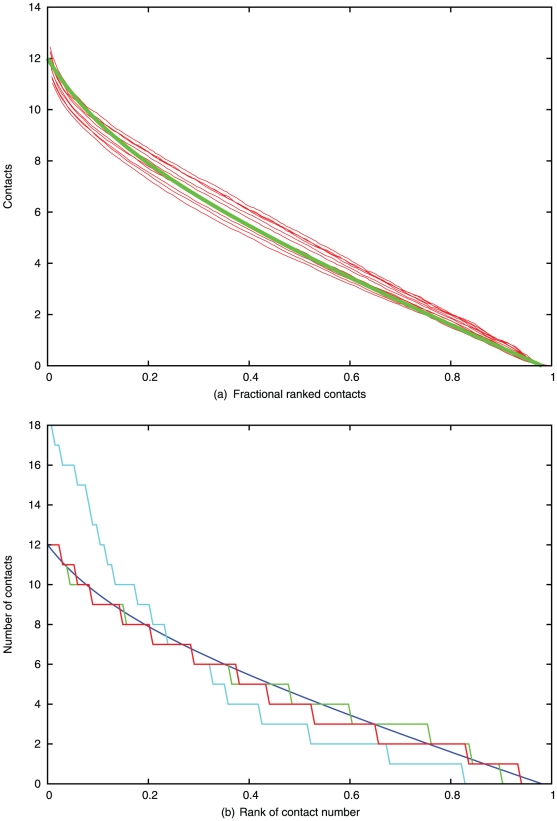
Number of contacts per residue. (*a*) The number of pseudo-centroid contacts is plotted against the fractional rank of the residues in the protein where 0 is most dense and 1 least dense. The red lines each represent data from the SCOP40 database in ten length bins spanning 100 to 200 residues. The green line is a fitted curve (described in the text). (*b*) Predicted contacts for 1f4p before correction (cyan) and after correction (red) are plotted along with the observed contacts (green) and the theoretical curve from part *a* (blue).

For SCOP40 domains in the range 100–200, the packing interactions were also broken down into the number of interactions per residue. When plotted against the fractional rank of the number of contacts (0 = most, 1 = least), the data lie almost on a straight line which is relatively independent of the protein size. (Figure 2).

While the data in Figure 2 could be reasonably approximated by a straight line, the sharp up-turn for the most densely packed residues can be better captured by including a reciprocal component: *R* = 8(1−*N*)+1.0(1/(*N*+0.2)−1), where *N* is again the number of residues. (Plotted as the green line in Figure 2).

**Figure 3:**
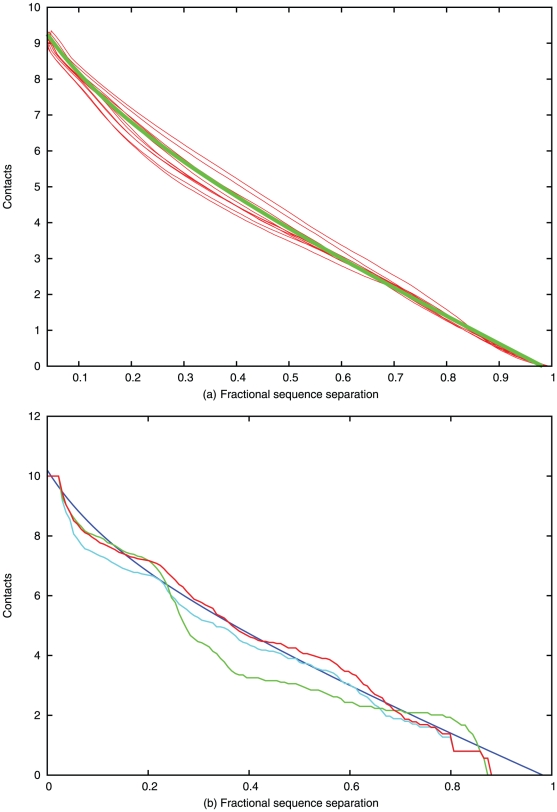
Number of contacts with sequence separation. (*a*) The number of pseudo-centroid contacts is plotted against the fractional sequence separation of the pair with 0 = adjacent to 1 = terminal residues. The red lines each represent data from the SCOP40 database in ten length bins spanning 100 to 200 residues. The green line is a fitted curve (described in the text). Note: it is coincidental that this curve is similar to that plotted in Figure 2. (*b*) Individual data for 1f4pA plotted using the same colours as Figure 2.

Residue packing was also analysed in terms of the sequential separation of the pair for the SCOP40 data (Figure 3). When plotted against the fractional ranked sequence separation (0 = adjacent, 1 = termini), an almost linear relationship is again found which also has a sharp up-turn as the pair spacing becomes small. The same functional form can be used to model these data, giving the relationship: *S* = 7(1−*N*)+0.8(1/(*N*+0.2)−1). (Plotted as the green line in Figure 3).

#### Application to predicted contacts

The quantities defined in the previous section (*P*,*R*,*S*) were used to refine the distribution of values in a predicted contact map as described in the Methods section. In this subsection we show the application of these constraints to the contacts predicted for the flavodoxin test protein (PDB code: 1f4pA).

In Figure 2(b) the observed contacts for 1f4pA, plotted in green, can be seen to be a close fit to the theoretical line derived from the SCOP40 data (blue). By contrast, the unrefined contacts contain many over-packed residues (cyan) with a maximum of 18 contacts. After correction (red) the distribution has been reduced to fit the theoretical curve, giving a close match to the observed data. When analysed by sequence separation, the observed contacts for 1f4pA, plotted in green in Figure 3(b), are a reasonable match to the theoretical distribution, as are the predicted contacts (cyan) which required only minor correction (red).

**Figure 4:**
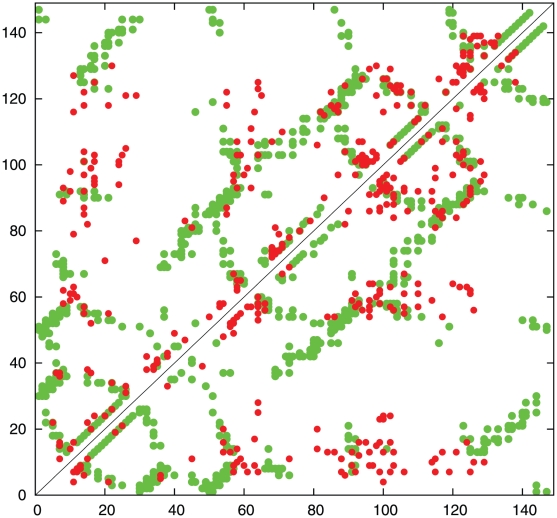
Contact map for 1f4pA showing predicted contacts before re-balancing (lower-right) and after correction (upper-left). Pseudo-centroid contacts under 8 Å are plotted in green.

The effect of these corrections on the contact map are compared in Figure 4 where the corrected contacts are plotted in the top left and the original contacts lower right of the map in red, against a background of observed contacts (green).

### Fold recognition

Each protein in the test-set (see Methods section) was run through the automated PLATO server [8], [9] to generate a large set of decoy models. (Strictly, these models are not all decoys as they include, typically 20 or more, folds that correspond to the native fold. For ease of reference, however, we will refer to them all as decoys or simply models.) The method was then modified by including the predicted SSE contacts to score the protein fold topologies generated at the low-level combinatorial stage, before any α-carbon coordinates are generated. The weaker scoring models are rejected at this stage allowing not only a greater search depth but also resulting in many fewer models. (See Table 1: “*number of decoy models*” column).

**Table 1:** Fold recognition over the test decoy sets.

	number of		basic method		DC method		DC best
PDB	decoy models		true folds		true folds		top model
code	basic	DC method	top 5	top 10	top 5	top 10	RMS (Å/ca)
2trx	16768	12526 (5046)	0	0	5 (1)	8 (4)	6.55/105
3chy	7015	700 (388)	1	2	5 (5)	10 (8)	5.70/121
1f4p	4243	3896 (991)	0	0	3 (5)	6 (8)	6.73/134
5p21	20169	5196 (1655)	2	4	4 (4)	4 (4)	8.70/158

**Figure 5:**
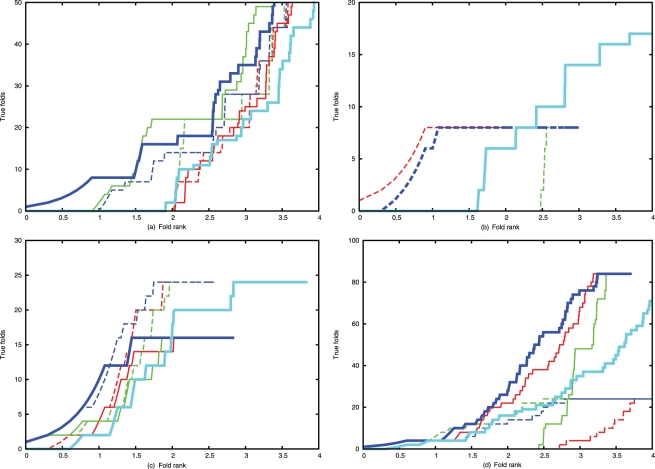
[parts: (a) 2trxA (b) 1f4pA (c) 3chyA (d) 5p21A]: True folds against (log) rank. The cumulative total number of true folds is plotted against the log(rank) of the model in the ranked list of decoys, up to a maximum of 10,000 models (4). As less than this number were sometimes constructed, the plots can end in ‘mid-air’. The result for the basic PLATO method is plotted in bold cyan. The models constructed by the contact augmented method were ranked by three scores: red, using just the basic PLATO score; green, using just the predicted contacts, and blue, using their combined score. The plots in dashed lines are the results after re-balancing the contact matrix with the structural constraints described in the text, using the same colour assignments.

Of the five proteins considered, one (1cozA) did not produce models and is not tabulated. For each of the other proteins (PDB) the number of decoys constructed by the basic PLATO method is tabulated in the leftmost column along with those using the PLATO method with direct contact (DC) information and, in parenthes, the number after applying structural constraints to the contact matrix. The computer execution time is roughly proportional to these numbers. The number of true folds (defined by topology string) found in the top 5 and top 10 ranked positions is tabulated for the basic PLATO method and the DC augmented method ranked by the PLATO score and the DC score combined as their geometric mean as used previously (with the arithmetic mean in parenthes). The number of hits over larger subsets is more easily seen in the plots in Figure 5. In the rightmost column, the root mean square (RMS) deviation was calculated over the number of residues (CA atoms) shown in parentheses for the top model. These values are slightly higher than some reported previously as the current models were not selected using residue-level contact data.

Using the uncorrected contacts, no models were generated for two of the proteins (1f4pA and 1cozA). As the unbiased PLATO server had generated good models for both proteins with the correct topology and RMS deviations around 5 Å, albeit low ranking, the limitation was not in the model generation but rather their scoring using the predicted contact data. For both these proteins, the contact map was refined as described in the previous section to correct for structural inconsistencies and the contact-biased PLATO server rerun. This now produced high ranking correct models for 1f4pA but still none for 1cozA. Examination of the raw data for 1cozA suggested that this should not be unexpected as they contain many false pairs.

As previously [6], the results for each protein were assessed by the position of models with the correct native fold (true fold) in the ranked list. As the root-mean-square deviation (or other measures) between models is unreliable [10] this was quantified unambiguously using topology diagrams encoded as a simple coordinate framework, called “topology strings” (see [6], [9] and papers cited therein for a definition). The ranks of the true folds were visualised by a simple ROC-like plot in which the cumulated number of true folds is plotted against the log of their rank. (Figure 5). The results of our previous study, which re-ranked the α-carbon models after construction, were summarised by whether any true fold had made it into the top-10 positions. By contrast, the current results are summarised by how many true folds are included in the top-10. (Table 1).

## Discussion

We have shown in this work that the application of predicted contacts derived from mutual information, can be applied with improved effect to the combinatorial fold generation level of the PLATO model construction method compared with previous results where it was applied only as a post-filter [6]. This entailed using the predicted contacts at the level of secondary structure elements (SSEs) rather than at the residue level. On the one hand, this had the advantage that the evaluation of the contacts did not depend on the exact phasing of the SSEs (that is, on the orientation of an α-helix around its axis or the up/down register of strands in a β-sheet) but, on the other hand, evaluation at the secondary structure level introduced the complication that, in the absence of a structure, the sequence of SSEs is not known. To overcome this, we relied on the many secondary structure variations generated by PLATO to include something close enough to the true assignment. However, it can be noted that the evaluation of pairwise contacts at this level does not use the nature of the SSE (α or β) but only the end-points and to make the method less sensitive to these, we also used part of the flanking loops at half-weight.

In our previous work it was also apparent that the predicted contact maps included features that were inconsistent with a folded protein structure. Often, the number of side-chains predicted to pack with a residue would exceed what was physically possible or the number of near neighbours in sequence would exceed what was compatible with the expected extension of the protein chain. In the past these problems have been encountered in the construction of protein models using distance geometry (DG) based on predicted distances [11], [12]. In that approach, inconsistent combinations of distances were gradually relaxed by repeated application of triangle-inequality balancing. However, with the current contact-based data, it would be necessary to propagate the contacts consistently throughout the matrix to provide a full set of distances that could then be embedded in 3D. We adopted a simpler approach in which the values were directly modified to conform to bulk properties derived from the SCOP40 database without concern for their detailed interdependence.

The combination and application of these new structural constraints greatly improved the selection of the true fold from the collection of decoy models constructed by PLATO. In our previous study on the same test set carried out before the introduction of these features [6], the application of predicted contact information was able only to lift the top rank of the true fold into the top 10 in four of the five proteins. In the current results, four of the five proteins have a majority of true folds in the top 5 and, on average, more than half of the top 10 positions consist of true folds. This is sufficient to unambiguously identify the correct fold for each set, based on sequence data alone. The odd-protein-out was 1cozA which, with its terminal helix unpacked, has never been predicted well. Nevertheless, the true fold did exist in the PLATO models but was not brought to the fore by the predicted contact data. A positive aspect of this negative result is that the poorly predicted contact constraints resulted in the rejection of all models so at least an incorrect model was not presented as a possible choice. It has often been pointed out in the CASP exercise how important it is to be able to make no prediction rather than a wrong one.

The use of predicted contacts from mutual information has provided powerful constraints on the selection of the correct fold against a background of well constructed decoys. Most importantly, no direct structural information has been used at any point in any of the stages of this method. Unlike methods that use fragments drawn from the protein structure database, all the decoy models constructed by PLATO derive from abstract theoretical constructs [13] and their elaboration into α-carbon models is based only on general principles of protein structure. Similarly on the sequence side, the alignments and mutual information were calculated without structural reference and only the predictions made by PsiPred within PLATO may contain a hint of structural memory in their neural-nets. However, given the quality and variability of the predictions, this does not seem likely.

The algorithmic change introduced in this work to the combinatorial method resulted in a more efficient search of the fold tree but made little difference in computational time requirements since this stage of the process is very fast (seconds) compared to the construction of the full α-carbon models which takes around one hour on a small linux cluster. However, it should allow progression to larger proteins where the combinatorial fold-space search can become significant. At the sizes where this can occur, however, the proteins will generally be multi-domain so it will firstly be necessary to use a domain identification algorithm as the ideal Forms used by PLATO assume a single domain. In this work we have focused on the βα class of protein. Although we have ideal forms of the all-β class and a limited set for the all-α, the smaller beta strands introduce greater uncertainty into the predictions and will be considered at a later stage.

All the aspects used in the current method could be further refined, however, there are issues of a more fundamental nature in the calculation of the underlying mutual information values that require more urgent attention. In particular, it is not clear that a simple method of sequence weighting is either optimal or needed and the treatment of gaps in the mutual information calculation, and phylogenetic structure in general, remains problematic. In this work we have shown only that a great improvement can be obtained over our previous implementation by incorporating a few simple structure-based features. We believe that with some improvement in the underlying raw data, including sequence alignment and direct contact calculation, the current method will provide a base from which to extend towards larger proteins or smaller families or, ideally, both together.

## Methods

**Figure 6:**
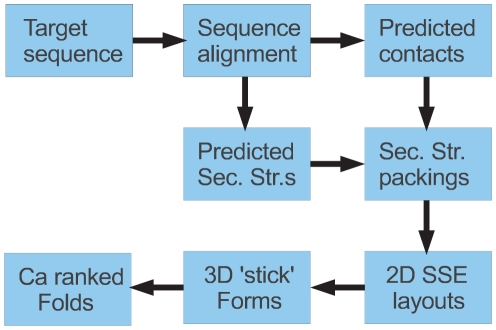
Overview of decoy model construction. The **Target sequence** to be predicted is matched against the sequence database to generate a multiple **Sequence alignment** which is used both to predict secondary structure (**Predicted Sec. Str.s**) and residue contacts (**Predicted contacts**). These two derived data sets are combined to estimate pairwise packing interactions at the secondary structure element (SSE) level (**Sec. Str. packings**) which are used in the PLATO method firstly to select the structural class of the protein via **2D SSE layouts** of the secondary structures. The corresponding stick models (**3D ‘stick’ Forms**) provide the framework over which different protein folds are combinatorially generated with pairings of secondary structures being evaluated by their predicted packing score. The ‘stick’ folds are then constructed at the residue (α-carbon) level giving the final set of **Ca ranked Folds**.

An overview of the methods described in the following sections can be found in Figure 6

### Protein Data

#### Test-set description

For test data, five βα-class proteins were selected, which for comparative purposes, have been well studied previously both as targets for protein structure prediction [8] and as test examples for the analysis of correlated alignment positions [6].

Identified by their protein structure databank (PDB) codes (with the chain designated by the terminal upper-case character and the number of residues in parentheses), the proteins were:

1. 2trxA (108) — a thioredoxin with a typical glutaredoxin fold. The protein contains the unusual topological feature of a helix located in a loop between two antiparallel β-strands. This helix usually is poorly predicted by the secondary structure prediction methods and often is modelled as a loop giving a larger RMS value when compared to the PDB structure for a protein of this size.
2. 1cozA (126) — a chorismate mutase with a mini-Rossman type fold plus a carboxy-terminal helix that packs across to the opposing monomer in this dimeric structure. In the definition of the correct fold, this terminal helix was considered correct if it packed back onto the domain either in an antiparallel or parallel connection, with the latter being a better approximation to the native structure. This protein has quite variable secondary structure predictions making it a difficult target.
3. 3chyA (128) — the chemotaxis Y protein (CheY). A compact protein with a flavodoxin-like fold that generally predicts well using just secondary structure prediction methods, or 3D prediction both with and without correlated mutation data.
4. 1f4pA (148) a classic (short-chain) flavodoxin. Although this protein has the same basic fold as 3chyA, the secondary structure elements are quite distinct both in size and packing and the two proteins are not even remotely homologous.
5. 5p21A (166) the Ras p21 G-protein which, although Rossmann-like, has a unusual embellishment of the edge of the domain comprising a parallel α-β connection leading into a long β-hairpin.

#### Decoy construction

Decoy models were constructed as described previously using the PLATO server that uses ideal Forms [13] to combinatorially generate thousands of folds that are then made into realistic α-carbon models [8], [9].

So that models and native structures can be treated equally, residue contacts were assessed as the distance between pairs of pseudo-centroid positions generated from the α-carbon coordinates by placing a point 2 Å along the bisector of the virtual β-carbon bond angle (on the obtuse side). This position lies close to the beta and gamma carbons of the side-chain when in the common trans conformation. For glycine, the α-carbon position was not used, as the models represent the variety of amino acids found in the multiple sequence alignment at each position.

#### Multiple sequence alignments

Multiple sequence alignments were taken directly from the PFAM database [5]. Since each family has a target structure corresponding to a single sequence entry, only positions in the alignment that correspond to un-gapped positions in the target sequence were considered.

As many of the PFAM families are very large and contain highly similar sequences, a simple and fast reduction was made by skipping any entry that was more than 95% identical with the preceeding entry or contained more than 20% of gapped positions, with both percentages calculated over the un-gapped positions of the target protein. This typically led to a 50% reduction in the number of sequences giving the following numbers for each family (with their PFAM identifier in parentheses):

1. 2trxA (PF00085) 12593 3692
2. 1cozA (PF01467) 6390 2996
3. 3chyA (PF00072) 75322 44072
4. 1f4pA (PF00258) 4607 2080
5. 5p21A (PF00071) 10390 4730

### Mutual Information (MI) Calculation

#### Raw MI score

We followed a standard calculation for the mutual information (*M*) between two positions in a multiple sequence alignment (*i* and *j*) as the difference in the Shannon entropy (*S*) between the sum of the individual positions 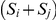 and their joint entropy 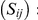


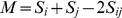
(1)


The entropy for a single position was calculated as the sum:
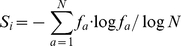
(2)


Where 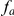 is the frequency of amino acid *a* in the aligned column of residues at position *i*. The sum is over the amino acid alphabet size (*N*) which was 21 (and the log in base 21) as an alignment gap was treated as an additional amino acid type. To prevent the log term becoming undefined when any amino acid is absent (log of 0), a pseudo-count was included in *f* as the equivalent of one amino acid evenly distributed over every entry in the column:
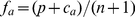
(3)


Where 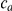 is the count of amino acid *a* over the *n* sequences at each aligned position and *p* is the pseudo-count which is 1/*N* for an alphabet of size *N* (i.e.: *p* = 1/21). When *n* is large, the entropy approaches 0 for a fully conserved position and 1 for the evenly distributed occurrence of every character irrespective of the alphabet size.

The entropy for a pair of positions is calculated as for a single position except that the alphabet is now the 441 amino acid pairs (21×21). Maintaining the identity of the two positions (*i* and *j*) this corresponds to:
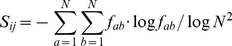
(4)


Where 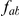 is the frequency of the amino acid pair *a* and *b*, including a pseudo-count as above but now with 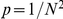. As above, the range of 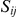 is between 0 and 1 which means that the mutual information (*M* in Equ*^n^*.1) also falls in this range.

#### Normalised MI score

Various methods have been used previously to normalise the MI with the simplest being to normalise by the joint entropy as:
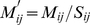
(5)


Following the nomenclature of [14], *M*′ will be referred to as MIr.

The same group developed a more complex normalisation using an estimated background based on the mean row 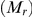 and column 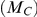 MI values relative to the overall mean MI 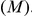 These quantities were combined either geometrically 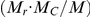 or arithmetically 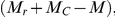 giving two scores: APC and ASC, respectively. Either background score can then be subtracted from the raw MI score to generate two normalised MI values: MIp and MIa being the product and additive alternatives, respectively.

### Direct Information (DI) Calculation

#### Inverse covariance matrix

The mutual information between a pair of positions contains indirect contributions from all their neighbours. Attempts to extract the direct contribution from the indirect have previously used a physics based [15], [16] or a Baysian based [17] approach. However, a simpler method has been employed to the equivalent problem of identifying direct from indirect interactions in protein networks [18] based on a property of the inverse covariance matrix called the partial correlation.

#### The generalised inverse

The covariance matrices derived from the standard MI can be poorly conditioned (i.e., may be singular) but are not particularly large, having only the rank of the number of positions in the alignment. (Typically, a few 100 square for small to medium sized proteins). A robust solution of their inverse can be obtained from the Moore-Penrose generalised inverse and a solution of this can be obtained from the singular value decomposition (SVD) of the matrix. (See Supplementary Information File S1).

From the components of the inverse matrix, the partial correlation coefficients, or direct information, (*D*) can be obtained as:
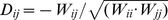
(6)where **W** is the inverse of the normalised MI matrix (**M**). An explanation of this derivation is given in the Supplementary Information File S2.

### Structural Constraints

The normalisation methods described above are largely concerned with the statistical properties of the interaction matrix with no account being taken that values in the matrix should represent a three-dimensional point-set. In general, given a matrix of pairwise distances for a point set, or a matrix of quantities that can be related to a distance, properties can be extracted from the matrix that have either a local or global correspondence to the physical object that they represent. This problem has previously been addressed in the context of protein structure prediction from sequence using distance geometry [11], [12] and a similar approach, based on cumulative distributions, will be used and adapted to deal with a limited set of predicted contacts rather than a full set of pairwise distances.

#### Number and distribution of contacts

For a given cutoff, either on distance or some corresponding score, the most immediate consequence is the total number of contacts that arise. This can be broken down into contacts per residue, resulting in a distribution of the number of contacts made for each residue. This distribution can then be compared to that obtained from known structures. A set of contacts can also be viewed in relation to their distribution in the sequence. A simple measure for this is the contact order measure [19] and although this was considered, reduction to a single number is too simple and instead the distribution of contacts with sequence separation was analysed.

In the light of these statistics, corrections were applied to re-balance the contact matrix. If any position (*i*) had more than the maximum estimated number of contacts, then starting with the worst violation, the matrix value 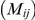 for each neighbour (*j*) was reduced by a small fraction (typically, 0.5%) and the process repeated for up to five cycles or until no violations remained. The overall balance of the matrix between sequentially local and distant contacts was corrected more directly by scaling each matrix value with a Gaussian function:
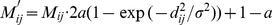
(7)where 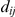 is the sequence separation between positions *i* and *j*, *a* is the size of the correction and σ is the range from the diagonal (like the standard deviation in the normal distribution). This was kept fixed at 100 residues whereas *a* was varied. Note that a positive value for *a* increases distant contacts and a negative value diminishes them.

#### Secondary structure scores

The linear nature of secondary structure elements (SSEs) introduce local correlation amongst the predicted contacts, creating clusters that align with the diagonal for parallel interactions and orthogonal to the diagonal for antiparallel packing. Some of the noise can be averaged from the predicted values using these patterns but only if it is firstly known where the SSEs are located. For this we use the predicted locations of the SSEs as described previously for the PLATO method. However, as there will be some uncertainty in the location of the SSEs, we included up to three residues either side of the SSE, up to half way towards the next SSE. These flanking residues were weighted by half in the following summations.

For a pair of SSEs, *i* and *j*, the total interaction strength 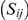 was the weighted sum of all the DC values in the sub-matrix corresponding to two SSEs and their flanking regions. A weighted centroid was calculated and used to divide this sub-matrix into quadrants over which (weighted) sub-sums 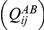 were taken, where *AB* can be: *NN*, *NC*, *CN* and *CC* for the sums on the amino-terminal side (*N*) and the C-terminal side (*C*)of the centroid. Parallel interactions will have higher *NN* and *CC* sums and antiparallel interactions higher *NC* and *CN* sums. A score 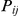 (for polarity) to reflect this was:
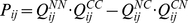
(8)giving a positive value for parallel and a negative value for antiparallel interactions.

#### Secondary structure packing

To compare the interaction scores devised in the previous subsection with a real or model protein structure we require an equivalent score at the SSE level. This was taken as a measure of interaction between the line segments along the axes of the secondary structures [20] with SSE definitions calculated in the same way for models and native structures [21]. The interaction measure was based on the overlap area of the line segments but summed as the reciprocal of the distance between the lines, which had previously been found to be a good approximation the interaction of SSEs as measured by changes in solvent accessible surface area [22]. This measure, referred to as the Reciprocal Overlap Area (ROA) was modified to account for screening by setting it to zero for SSEs on opposite sides of a β-sheet or (severely) damped as 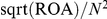, where *N* is the adjacency of the SSEs in a layer, designated as 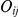 for a pair of SSEs.

An overall interaction score was calculated as a combination of an un-oriented interaction (to account for orthogonal or poor orientation discrimination) and an orientated component:
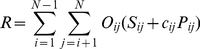
(9)where 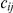 is the cosine of the dihedral angle between the line segments of SSEs *i* and *j*. Note that both *c* and *P* are signed so the overall score (*R*) will include negative contributions.

## Supporting Information

File S1 Implementation notes on the Moore-Penrose generalized inverse.
(TXT)

File S2 Explanatory notes on the partial correlation calculation.
(PS) Click here for additional data file.

## References

[1] Pollock DD, Taylor WR (1997). Effectiveness of correlation analysis in identifying protein residues undergoing correlated evolution.. Prot Engng.

[2] Taylor WR, Jones DT, Green NM (1994). A method for α-helical integral membrane protein fold prediction.. Prot Struct Funct Genet.

[3] Pollock DD, Taylor WR, Goldman N (1999). Coevolving protein residues: maximum likelihood identification and relationship to structure.. J Molec Biol.

[4] Bartlett GJ, Taylor WR (2008). Using scores derived from statistical coupling analysis to distinguish correct and incorrect folds in *de-novo* protein structure prediction.. Proteins: struct funct bioinfo.

[5] Bateman A, Coin L, Durbin R, Finn RD, Hollich V (2004). The pfam protein families database.. Nucleic Acids Res.

[6] Sadowski MI, Maksimiak K, Taylor WR (2011). Direct correlation analysis improves fold recognition.. Compu Biol Chem.

[7] Murzin AG, Brenner SE, Hubbard T, Chothia C (1995). SCOP: a structural classification of proteins database for the investigation of sequences and structures.. J Molec Biol.

[8] Taylor WR, Bartlett GJ, Chelliah V, Klose D, Lin K (2008). Prediction of protein structure from ideal forms.. Proteins: struct, funct, bioinfo.

[9] Taylor WR, Hollup SM, MacDonald JT, Jonassen I (2009). Probing the “dark matter” of protein fold-space.. Structure.

[10] Hollup SM, Sadowski MI, Jonassen I, Taylor WR (2011). Exploring the limits of fold discrimination by structural alignment: A large scale benchmark using decoys of known fold.. Compu Biol Chem.

[11] Aszódi A, Taylor WR (1994). Folding polypeptide α-carbon backbones by distance geometry methods.. Biopolymers.

[12] Aszódi A, Taylor WR (1995). Estimating polypeptide α-carbon distances from multiple sequence alignments.. J Math Chem.

[13] Taylor WR (2002). A periodic table for protein structure.. Nature.

[14] Dunn SD, Wahl LM, Gloor GB (2008). Mutual information without the influence of phylogeny or entropy dramatically improves residue contact prediction.. Bioinformatics.

[15] Lapedes AS, Giraud BG, Liu L, Stormo GD (1999). Correlated mutations in models of protein sequences: phylogenrtic and structural effects.. Statistics in Mol Biol.

[16] Weigt M, White RA, Szurmantc H, Hochc JA, Hwa T (2009). Identification of direct residue contacts in protein-protein interaction by message passing.. PNAS.

[17] Burger L, van Nimwegen E (2010). Disentangling direct from indirect co-evolution of residues in protein alignments.. PLoS comp biol.

[18] Friedman J, Hastie T, Tibshirani R (2008). Sparse inverse covarience estimation with the graphical lasso.. Biostatistics.

[19] Plaxco KW, Simons KT, Baker D (1998). Contact order, transition state placement and the refolding rates of single domain proteins.. J Molec Biol.

[20] Taylor WR, Thornton JM, Turnell WG (1983). An ellipsoidal approximation of protein shape.. J Molec Graphics.

[21] Taylor WR (2001). Defining linear segments in protein structure.. J Molec Biol.

[22] Taylor WR (2002). Protein structure comparison using bipartite graph matching.. Mol Cell Proteomics.

